# Supplementary material for: Resolving the full spectrum of human genome variation using Linked-Reads
Source: Genome Res. 2019 Apr;29(4):635–45. doi: 10.1101/gr.234443.118 (PMC6442396; doi:10.1101/gr.234443.118)
Supplement: Supplemental Material [file supp_gr.234443.118_Supplemental_longranger-2.2.zip › longranger-2.2.tar.bz2!/lr2.2-public/lib/go/src/code.google.com/vitess/docs/user-guide/introduction.html]

Vitess / Vitess User Guide - Introduction


Toggle navigation

- Overview
- Guides
- Reference
- Blog
- GitHub

- #### Overview

  - What is Vitess
  - Scaling MySQL with Vitess
  - Key Concepts
- #### Getting Started

  - Run Vitess on Kubernetes
    - Custom Docker Build
  - Run Vitess Locally
- #### User Guide

  - Introduction- Client Libraries- Backing Up Data- Reparenting
        - Schema Management
        - VSchema Guide
        - Schema Swap (Tutorial)
        - Sharding
          - Horizontal Sharding (Tutorial, automated)
          - Horizontal Sharding (Tutorial, manual)
          - Sharding in Kubernetes (Tutorial, automated)
          - Sharding in Kubernetes (Tutorial, manual)
        - Vitess Sequences
        - MySQL Server Protocol
        - Vitess and Replication
        - Update Stream
        - Row Based Replication
        - Topology Service
        - Transport Security Model
        - Launching
          - Scalability Philosophy
          - Production Planning
          - Server Configuration
          - 2PC Guide
          - Troubleshooting
        - Upgrading
- #### Reference Guides

  - Vitess API- vtctl Commands
- #### Other Resources

  - Presentations- Blog- Roadmap
- #### Contributing

  - Contributing to Vitess- GitHub Workflow- Code Reviews
- GitHub

# Vitess User Guide - Introduction

- #### Overview

  - What is Vitess
  - Scaling MySQL with Vitess
  - Key Concepts
- #### Getting Started

  - Run Vitess on Kubernetes
    - Custom Docker Build
  - Run Vitess Locally
- #### User Guide

  - Introduction- Client Libraries- Backing Up Data- Reparenting
        - Schema Management
        - VSchema Guide
        - Schema Swap (Tutorial)
        - Sharding
          - Horizontal Sharding (Tutorial, automated)
          - Horizontal Sharding (Tutorial, manual)
          - Sharding in Kubernetes (Tutorial, automated)
          - Sharding in Kubernetes (Tutorial, manual)
        - Vitess Sequences
        - MySQL Server Protocol
        - Vitess and Replication
        - Update Stream
        - Row Based Replication
        - Topology Service
        - Transport Security Model
        - Launching
          - Scalability Philosophy
          - Production Planning
          - Server Configuration
          - 2PC Guide
          - Troubleshooting
        - Upgrading
- #### Reference Guides

  - Vitess API- vtctl Commands
- #### Other Resources

  - Presentations- Blog- Roadmap
- #### Contributing

  - Contributing to Vitess- GitHub Workflow- Code Reviews

## Platform support

We continuously test against Ubuntu 14.04 (Trusty) and Debian 8 (Jessie).
Other Linux distributions should work as well.

## Database support

Vitess supports MySQL 5.6,
MySQL 5.7,
and MariaDB 10.0.

### Data types and SQL support

In Vitess, database tables are like MySQL relational tables, and you
can use relational modeling schemes (normalization) to structure your
schema. Vitess supports both primary and secondary indexes.

Vitess supports almost all MySQL scalar data types.
It also provides full SQL support within a
shard, including JOIN statements.

Vitess does not currently support encoded protobufs or protocol buffer
querying. (The latter is also known as cracking.) Protocol buffers can
be stored as a blob in MySQL, but must be decoded and interpreted at
the application layer.

### Schema management

Vitess supports several functions for looking at your schema and
validating its consistency across tablets in a shard or across all
shards in a keyspace.

In addition, Vitess supports
data definition statements
that create, modify, or delete database tables. Vitess executes
schema changes on the master tablet within each shard, and those
changes then propagate to slave tablets via replication. Vitess does
not support other types of DDL statements, such as those that affect
stored procedures or grants.

Before executing a schema change, Vitess validates the SQL syntax
and determines the impact of the change. It also does a pre-flight
check to ensure that the update can be applied to your schema. In
addition, to avoid reducing the availability of your entire system,
Vitess rejects changes that exceed a certain scope.

See the Schema Management
section of this guide for more information.

## Supported clients

You can access your Vitess cluster using a variety of clients and
programming languages.

Vitess' service is exposed through a
proto3
service definition. Vitess supports gRPC,
and you can use the
proto compiler
to generate stubs that can call the API in any language that the
gRPC framework supports.

### Client libraries

Client libraries that support a richer set of functionality are
available for some languages. Client libraries help your application
to more easily talk to your storage system to query data.

The following table lists those
client libraries and other clients that Vitess supports.

| Type | Options |
| --- | --- |
| Client library | gRPC Go Java Python PHP |
| MapReduce | Hadoop input |

## Backups

Vitess supports data backups to either a network mount (e.g. NFS) or to a blob store.
Backup storage is implemented through a pluggable interface,
and we currently have plugins available for Google Cloud Storage, Amazon S3,
and Ceph.

See the Backing Up Data section
of this guide for more information about creating and restoring data
backups with Vitess.

Contact: vitess@googlegroups.com  **·**  
Announcements  **·**  
© 2017 Vitess powered by Google Inc
